# FBXO28 promotes proliferation, invasion, and metastasis of pancreatic cancer cells through regulation of SMARCC2 ubiquitination

**DOI:** 10.18632/aging.204780

**Published:** 2023-06-21

**Authors:** Songbai Liu, Peng Liu, Changhao Zhu, Rui Yang, Zhiwei He, Yongning Li, Ying Li, Xiaobin Fei, Junyi Hou, Xing Wang, Yaozhen Pan

**Affiliations:** 1 Guizhou Medical University, Guiyang 550000, Guizhou, China; 2 Department of Hepatic-Biliary-Pancreatic Surgery, The Affiliated Cancer Hospital of Guizhou Medical University, Guiyang 550000, Guizhou, China; 3 Department of Hepatic-Biliary-Pancreatic Surgery, The Affiliated Hospital of Guizhou Medical University, Guiyang 550000, Guizhou, China; 4 Department of Hepatobiliary Surgery, Shenzhen Key Laboratory, Shenzhen University General Hospital, Shenzhen 518055, Guangdong, China

**Keywords:** pancreatic cancer, FBXO28, ubiquitination, SMARCC2, proliferation

## Abstract

The E3 ligase F-box only protein 28 (FBXO28) belongs to the F-box family of proteins that play a critical role in tumor development. However, the potential function of FBXO28 in pancreatic cancer (PC) and its molecular mechanism remain unclear. In this study, we examined FBXO28 expression in PC and its biological role and explored the mechanism of FBXO28-mediated proliferation, invasion, and metastasis of PC cells. Compared with paracancerous tissues and human normal pancreatic ductal epithelial cells, FBXO28 was highly expressed in PC tissues and cell lines. High expression of FBXO28 was negatively correlated with the survival prognosis of patients with PC. Functional assays indicated that FBXO28 promoted PC cell proliferation, invasion, and metastasis *in vitro* and *in vivo*. Furthermore, immunoprecipitation-mass spectrometry was used to identify SMARCC2 as the target of FBXO28; upregulation of SMARCC2 can reverse the effect of overexpression of FBXO28 on promoting the proliferation, invasion, and metastasis of PC cells. Mechanistically, FBXO28 inhibited SMARCC2 expression in post-translation by increasing SMARCC2 ubiquitination and protein degradation. In conclusion, FBXO28 has a potential role in PC, possibly promoting PC progression through SMARCC2 ubiquitination. Thus, FBXO28 might be a potential treatment target in PC.

## INTRODUCTION

Pancreatic cancer (PC) is a digestive tract tumor with high malignancy, difficult early diagnosis, and limited treatment options. Importantly, it has a 5-year survival rate of less than 10% and is the fourth leading cause of cancer-related fatalities in the United States [1, 2]. Further, PC is predicted to become the second leading cause of cancer-related deaths in the United States by 2030 [3]. Given that patients with early-stage PC have no obvious clinical symptoms, more than 80% of patients have advanced stage at initial diagnosis [4], and more than 95% of the patients have metastases or will have metastases at follow-up [5]. Although there have been significant advances in PC therapy, surgical excision remains a fundamental modality of PC treatment [6]. However, PC patients have a very poor prognosis even after surgery owing to the high recurrence rate and highly aggressive nature of the malignancy [7]. Consequently, it is necessary to investigate the underlying molecular mechanism of PC so that it can be diagnosed early and treated effectively.

The ubiquitin proteasome system (UPS) is the most critical non-lysosomal protein degradation mechanism in eukaryotic cells [8]. Ubiquitin consists of 76 amino acids and is a highly conserved protein expressed in all eukaryotic cells [9, 10]. Ubiquitination is the covalent binding of ubiquitin to substrate proteins catalyzed by E1-activating enzymes, E2-binding enzymes, and E3 ligases [11] and is essential for the maintenance of intracellular homeostasis. (Skp1-Cullin1-F-box protein) E3 ubiquitin ligase performs a crucial role in several biological processes [12], including the cell cycle, transcription, translation, DNA damage, and repair, by promoting the ubiquitination of cellular substrates [13]. FBXO28 is a member of the F-box protein family, the cellular roles of which are not completely understood. Recent research has shown that abnormal FBXO28 expression is closely correlated with poor overall survival (OS) and prognosis in breast cancer [14].

SMARCC2 is an SWI/SNF subunit that plays a crucial role in the formation of tumors including colorectal cancer [15] and breast cancer [16]. Current data indicate that the SWI/SNF complex functions as a tumor suppressor, but its precise mechanism of action remains unknown [17]. The SWI/SNF complex uses ATP-dependent chromatin remodeling activity to mobilize nucleosomes, thereby enabling dynamic regulation of chromatin structure [18, 19]. The complex consists of series of highly conserved core subunits, including SMARCB1/INI1, SMARCA4/BRG1, SMARCC1/BAF155, and SMARCC2/BAF170 [20, 21]. Recent studies have shown that SMARCC2 inhibits Wnt/β-catenin signaling by mediating the expression of the oncogene C-MYC, thereby inhibiting the migration and invasion abilities of glioma cells [22]. Studies have found that SMARCC2 can be modified and regulated by phosphorylation and methylation. For example, activated nuclear RIPK1 mediates phosphorylation of SMARCC2, a vital component of the BAF complex, thereby promoting chromatin remodeling and transcription of specific pro-inflammatory genes [23]. Furthermore, the demethylase LSD1 demethylates methylated lysine residues in SMARCC1 and SMARCC2 to maintain the structural integrity of the SWI/SNF complex [24]. However, SMARCC2 is still relatively poorly studied in terms of ubiquitination modifications.

The goal of this study was to assess FBXO28 expression in PC and to explore its biological role. Our results revealed a possible underlying mechanism for FBXO28 in PC, in which it plays an oncogenic role by mediating the ubiquitination of SMARCC2 and promoting the degradation of SMARCC2 in PC cells.

## RESULTS

### FBXO28 is highly expressed in PC and associated with poor survival prognosis

**Figure 1:**
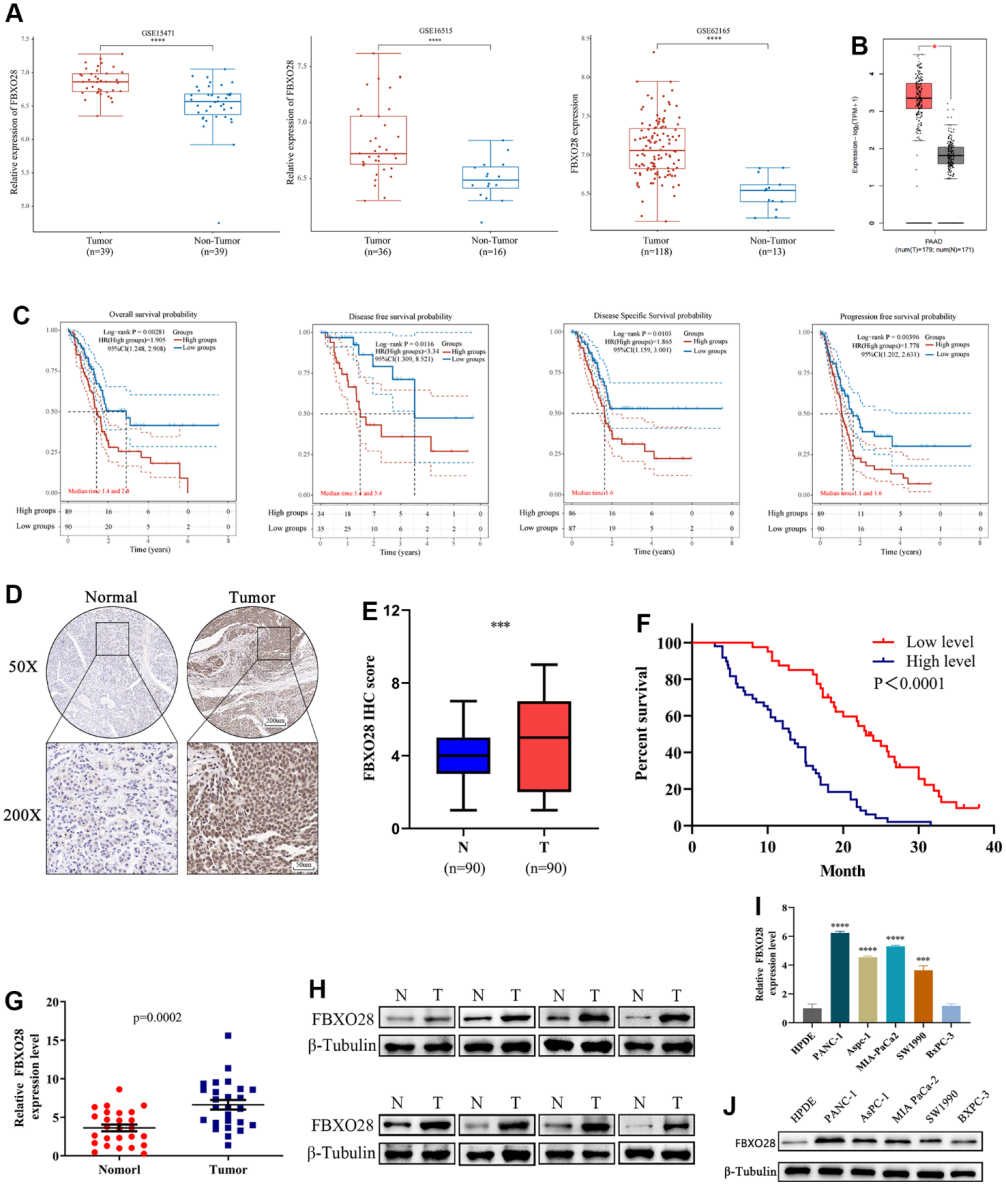
**FBXO28 is overexpressed in pancreatic cancer and is associated with poor prognosis.** (**A**, **B**) In the GSE15471, GSE16515, GSE62165, and GEPIA2 datasets, pancreatic cancer (PC) tissue specimens had substantially higher levels of FBXO28 expression than paracancerous tissue specimens. (**C**) Based on data from The Cancer Genome Atlas database, Kaplan–Meier analysis reveals that FBXO28 expression is associated with overall survival (OS), disease-free survival (DFS), disease-specific survival (DSS), and progression-free survival (PFS) in patients with PC. (**D**, **E**) Typical IHC images show high expression of FBXO28 in pancreatic cancer tissues (magnification: ×50, ×200). (**F**) Kaplan–Meier survival curves for high and low expression of FBXO28 in patients with PC. (**G**, **H**) FBXO28 expression in PC and paracancerous tissues detected by qRT-PCR and western blot. (**I**, **J**) FBXO28 expression in five PC cells was examined using qRT-PCR and western blot analysis, with β-tubulin serving as a control variable. ***P < 0.001, ****P<0.0001.

Gene Expression Omnibus (GEO) (GSE16515, GSE15471, GSE62165) and GEPIA2 dataset analyses showed that FBXO28 is highly expressed in PC (Figure 1A, 1B). Kaplan-Meier analysis of FBXO28 and OS, disease-free survival, disease-specific survival, and progression-free survival of patients with PC showed that the expression of FBXO28 was negatively correlated with survival and prognosis of PC (Figure 1C). We next compared 90 PC and paracancerous tissue samples using tissue microarray, and immunohistochemical (IHC) analysis revealed that FBXO28 expression is significantly higher in PC tissues than in paracancerous tissues (Figure 1D, 1E). For the association between FBXO28 expression and clinicopathological features, high FBXO28 expression was found to be significantly correlated with pTNM stage (P = 0.003), T stage (P = 0.033), and lymphatic metastasis (P = 0.001), but not with age, sex, tumor size, distant metastasis, and nerve invasion (Table 1). Survival analysis showed that patients with PC with high FBXO28 expression had considerably shorter OS than those with low expression (Figure 1F). Validation with quantitative real-time polymerase chain reaction (qRT-PCR) and western blot of 26 PC and paracancerous tissues showed that FBXO28 expression was significantly higher at the mRNA and protein levels in PC tissues than in paracancerous tissues (Figure 1G, H). We further verified the PC cell lines, and the results also showed that the expression of FBXO28 in PC cell lines was higher than that in human pancreatic ductal epithelial (HPDE) cells (Figure 1I, 1J). These experimental findings showed that FBXO28 is highly expressed in PC tissues and cell lines and is related to poor prognosis in patients with PC, indicating that FBXO28 may have a pro-cancer function in PC.

**Table 1:** The relationship between FBXO28 expression and clinicopathological characteristics in PC patients.

**FBX028 expression**						
**Clinical characteristic**		**Low**	**High**	**n**	***X^2^***	***p-Value***
Gender	Male	26	27	53	1.720	0.204
	Female	13	24	37		
Age (years)	<60	23	24	47	1.258	0.293
	≥60	16	27	43		
Tumor size (cm)	≤4	29	31	60	1.833	0.259
	>4	10	20	30		
pTNM stage	I-II	30	23	53	9.246	**0.003**
	III-IV	9	28	37		
T stage	I-II	25	20	45	5.475	**0.033**
	III-IV	14	31	45		
Lymph node metastasis	No	22	11	33	11.553	**0.001**
	Yes	17	40	57		
Distant metastasis	No	32	34	66	2.675	0.149
	Yes	7	17	24		
Vessel invasion	No	28	24	52	5.543	**0.031**
	Yes	11	27	38		
Nerve invasion	No	16	15	31	1.320	0.271
	Yes	23	36	59		
Histological grade	Low	14	21	35	0.259	0.666
	Middle-high	25	30	55		

### FBXO28 overexpression promotes PC cell proliferation *in vitro* and tumor growth *in vivo*

To investigate the function of FBXO28 in cells, we constructed FBXO28 overexpression and knockdown lentiviruses and validated the results using qRT-PCR and western blot (Figure 2A). Cell Counting Kit-8 (CCK-8) and EdU tests revealed that FBXO28 overexpression markedly increased the proliferative capacity of PC cells, whereas FBXO28 downregulation hindered PC cell proliferation (Figure 2B–2D). The cell clone formation assay showed that FBXO28 upregulation promoted PC colony-forming ability, whereas FBXO28 downregulation inhibited PC cell colony-forming ability (Figure 2E). Western blot analysis to understand the cell cycle effect of FBXO28 showed that FBXO28 upregulation increased Cyclin E1, cyclin-dependent kinase (CDK) 2, and CDK4 expression and decreased P27 expression (Figure 2F). In flow cytometry, FBXO28 upregulation promoted PC cell conversion from G1 to S phase, whereas FBXO28 downregulation resulted in the opposite (Figure 2G, 2H). To better elucidate the biological impact of FBXO28 on tumor development, we performed a subcutaneous tumor formation assay in nude mice. Tumor volume and weight were obviously higher in the FBXO28 overexpression group than in the control group, whereas they were markedly lower in the FBXO28 downregulation group (Figure 2I). IHC analysis of tumor tissue samples showed that FBXO28 upregulation enhanced the staining of the proliferation-associated genes Ki67 and proliferating cell nuclear antigen (PCNA), with a significantly higher proportion of positive cells in the FBXO28 upregulation group than in the control group. Meanwhile, FBXO28 downregulation markedly decreased the proportion of positive cells (Figure 2J). Consequently, FBXO28 may play an essential role in the proliferation of PC cells.

**Figure 2:**
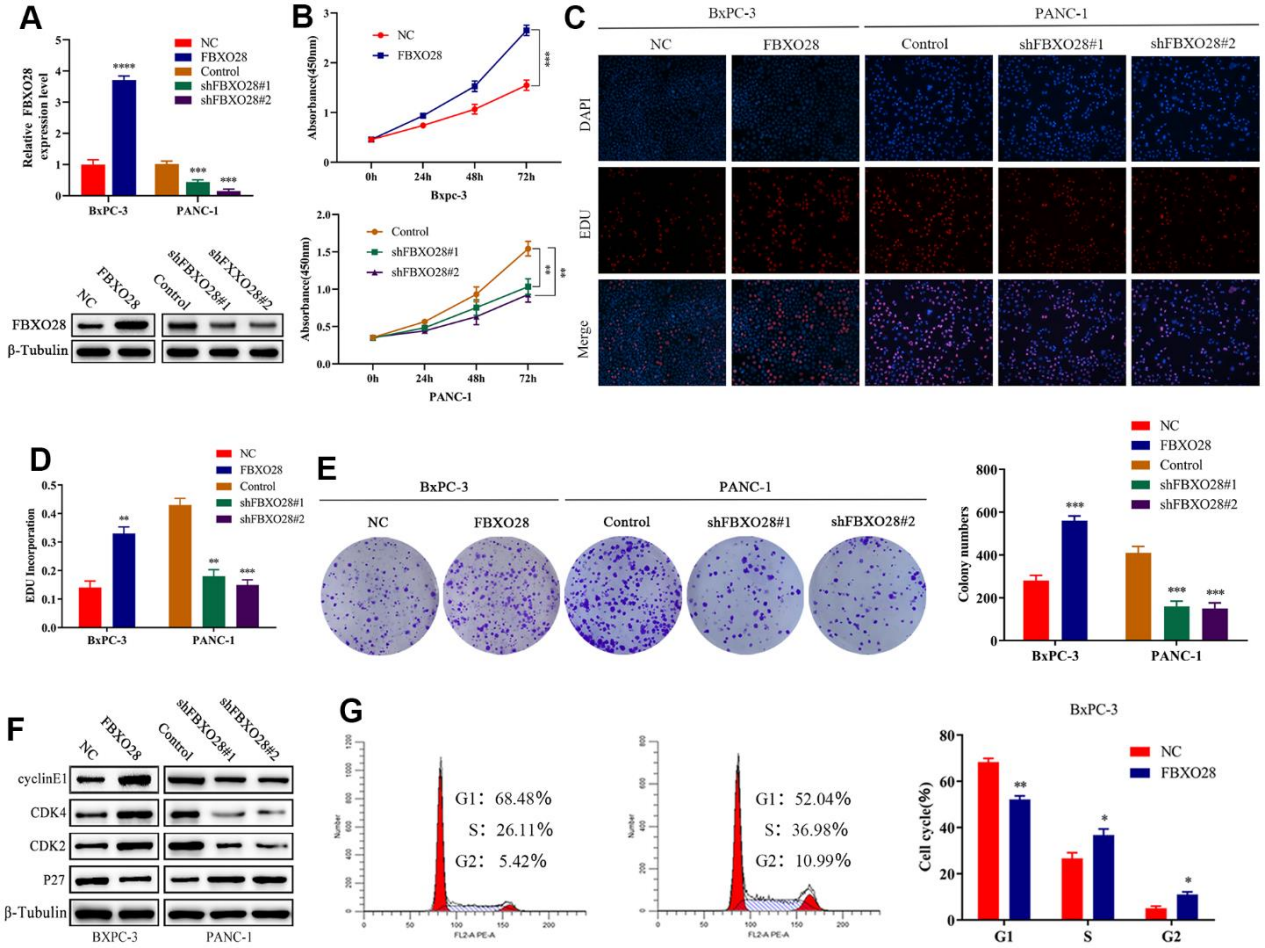
**FBXO28 overexpression increases pancreatic cancer cell proliferation.** (**A**) Lentiviral transfection to form stable cells (negative control [NC], FBXO28, Control, shFBXO28#1, shFBXO28#2) and qRT-PCR and western blot to verify transfection effectiveness. (**B**–**E**) Cell Counting Kit-8 (CCK-8), EdU, and clone plate experiments were used to identify the capacity of FBXO28 for cell proliferation and formation in pancreatic cancer cells. (**F**, **G**). **P < 0.01, ***P < 0.001, ****P < 0.0001.

**Figure 2:**
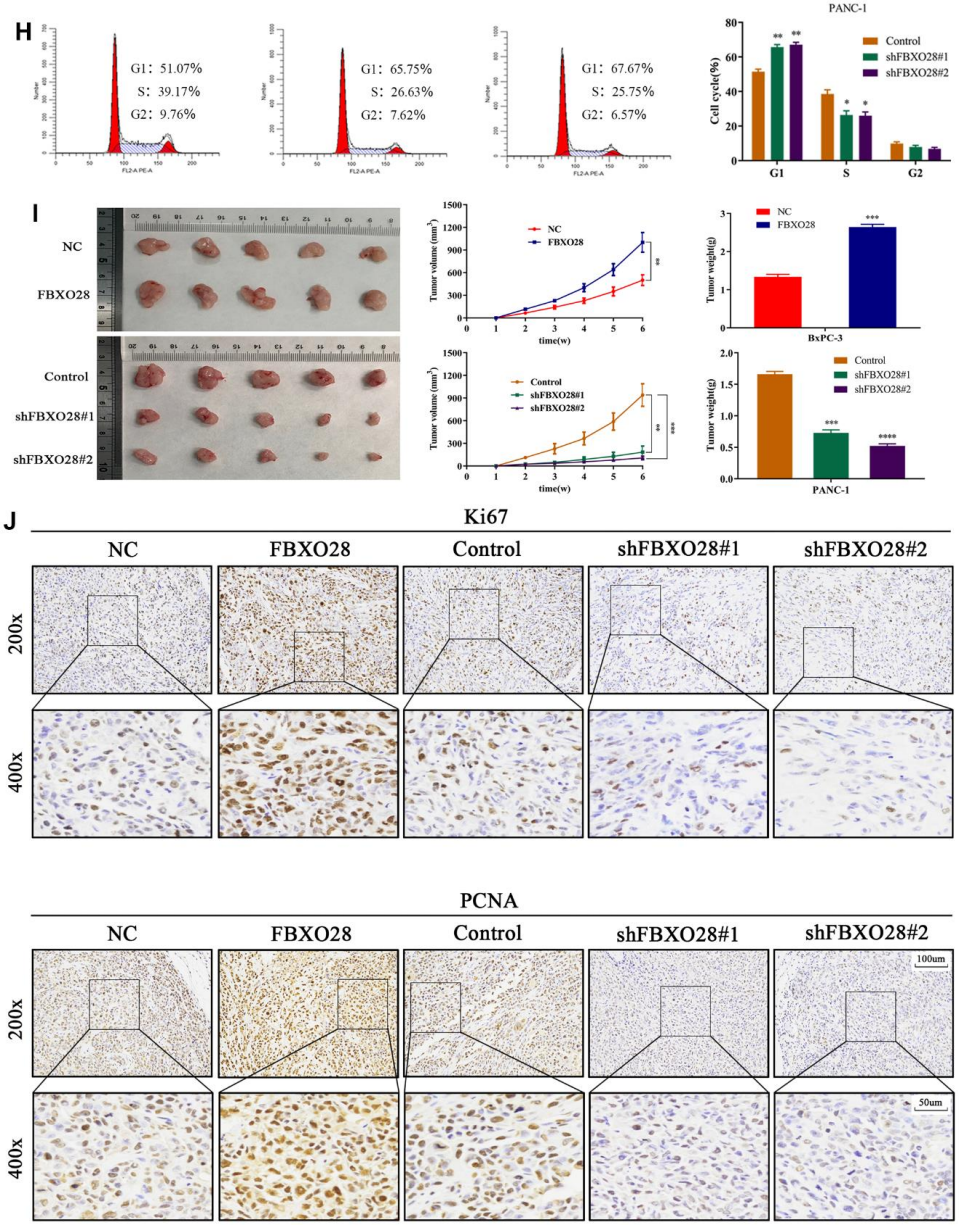
**FBXO28 overexpression increases pancreatic cancer cell proliferation.** (**H**) Western blot and flow cytometry to investigate the effect of FBXO28 on the cell cycle. (**I**) To construct a xenograft model, mice were injected subcutaneously with cells according to grouping, tumor volume was assessed weekly, the mice were euthanized after 6 weeks, and the tumors were resected and weighed. (**J**) Immunohistochemical (IHC) of mouse tumor tissues showing the protein expression of Ki67 and proliferating cell nuclear antigen (PCNA) (magnification: ×200, ×400). **P < 0.01, ***P < 0.001, ****P < 0.0001.

### FBXO28 overexpression promotes invasive migration of PC cells *in vitro* and tumor migration *in vivo*

**Figure 3:**
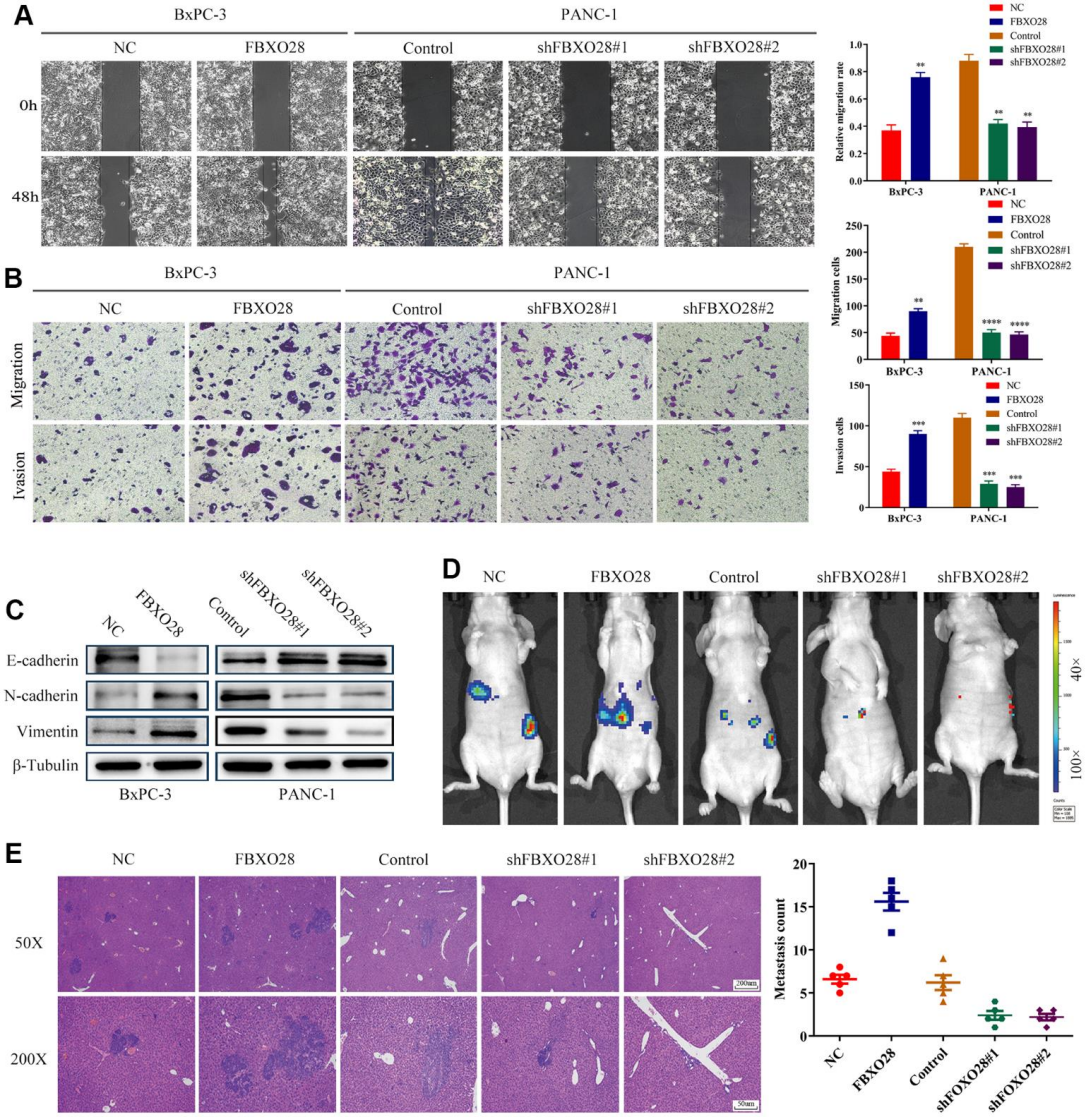
**Overexpression of FBXO28 promotes invasion and migration of pancreatic cancer cells.** (**A**) At 24 hours after scratching, the migratory capacity of PANC-1 and BxPC-3 cells was assessed using the wound healing assay. (**B**) Transwell assay evaluation of BxPC-3 and PANC-1 cell invasion and migration. (**C**) Western blot to verify the expression of FBXO28 and epithelial-mesenchymal transition (EMT)-related proteins. (**D**) The cells were injected into the spleen to establish a liver metastasis model in the mice according to grouping, and live imaging was performed. (**E**) Hematoxylin-and-eosin (HE) staining of liver metastases (magnification: ×50, ×200). **P < 0.01, ***P < 0.001, ****P < 0.0001.

To further explore the effect of FBXO28 on the migration and invasion of PC cells, we first verified it through wound healing experiments. Upregulation of FBXO28 enhanced the migration ability of PC cells, whereas downregulation inhibited the migration ability of PC cells (Figure 3A). Transwell experiments showed that the number of invasive migrating PC cells in the FBXO28 overexpression group was significantly higher than that in the control group. In contrast, the number of invasive migrating PC cells in the FBXO28 downregulation group was significantly reduced (Figure 3B). Experiments with epithelial-mesenchymal transition-associated proteins indicated that FBXO28 upregulation increased N-cadherin and vimentin expression and decreased E-cadherin expression, whereas FBXO28 downregulation produced the opposite effect (Figure 3C). Finally, the mouse liver metastasis model showed that FBXO28 overexpression significantly promoted metastasis *in vivo*, whereas FBXO28 downregulation inhibited metastasis *in vivo* (Figure 3D). Hematoxylin-and-eosin staining of liver specimens showed that the number and size of liver lesions were higher in the upregulated FBXO28 group than that in the control group. In contrast, in the downregulated FBXO28 group, the number and size of liver lesions were significantly lower than that in the control group (Figure 3E). These results suggested that FBXO28 promotes PC cell migration and invasion both *in vitro* and *in vivo*.

### SMARCC2 is a critical target of FBXO28

**Figure 4:**
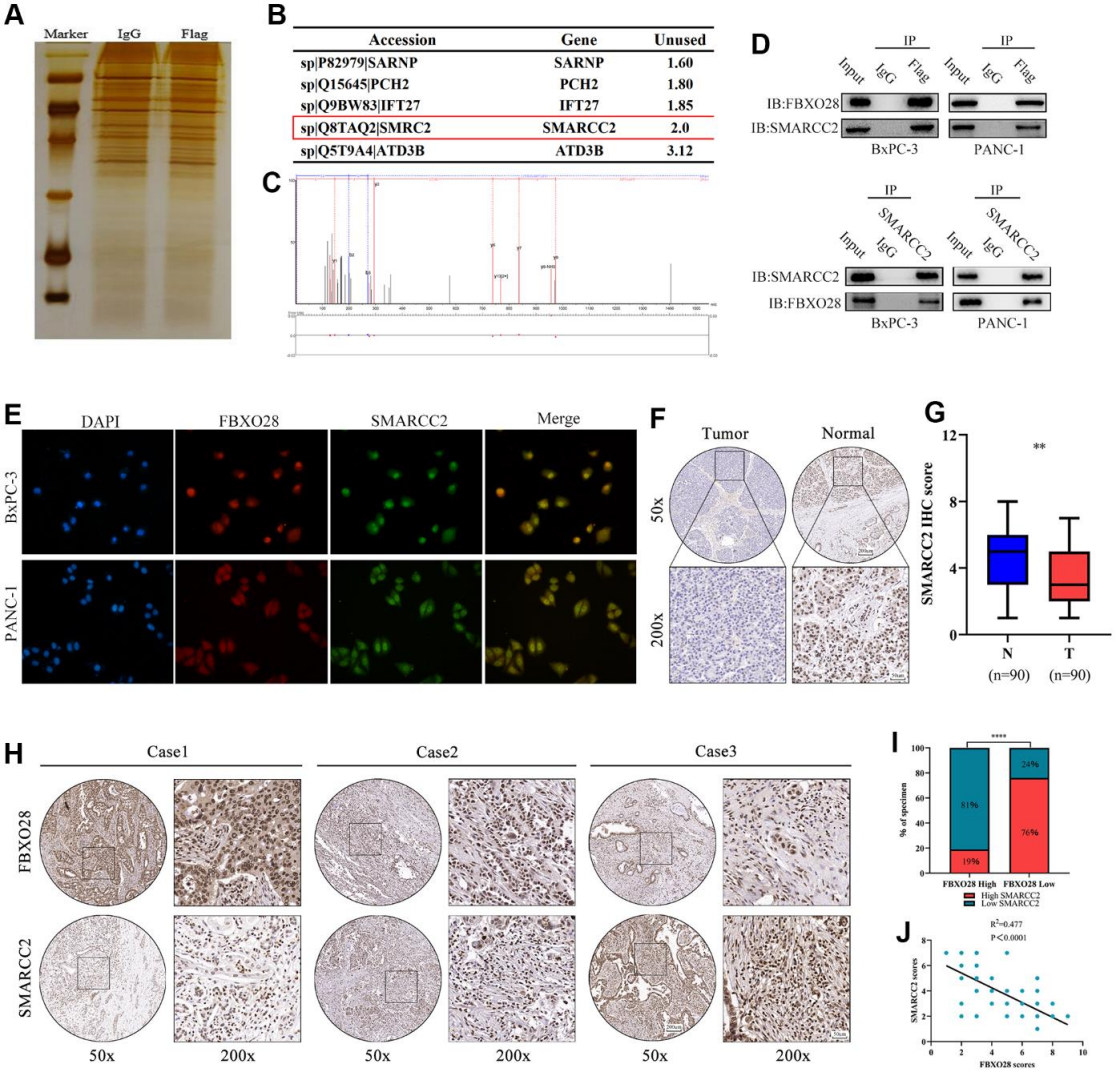
**SMARCC2 is a critical target of FBXO28.** (**A**) BxPC-3 cells with stable FBXO28 overexpression, as well as control cells, were subjected to sodium dodecyl sulfate-polyacrylamide gel electrophoresis and band staining for observation. Mass spectrometry was used to identify and isolate protein bands. (**B**) Liquid chromatography with tandem mass spectrometry (LC-MS/MS) analysis of five proteins co-precipitated with FBXO28. (**C**) Mass spectrometry revealed a unique peptide of FBXO28 identified by two-dimensional LC-MS/MS from protein lysates of anti-FBXO28 immunoprecipitated BxPC-3 cells. (**D**) The interaction between FBXO28 and SMARCC2 was confirmed using a co-immunoprecipitation assay, and western blots were performed on cell lysates. (**E**) Co-localization analysis of FBXO28 and SMARCC2 in the nucleus by immunofluorescence. (**F**, **G**) Typical immunohistochemical (IHC) image showing low expression of SMARCC2 in pancreatic cancer tissue (magnification: ×50, ×200). (**H**–**J**) Immune tissue co-expression revealed a negative association between FBXO28 and SMARCC2 expression within pancreatic cancer tissues (magnification: ×50, ×200). ****P < 0.0001.

To further explore the molecular targets of FBXO28 in PC, immunoprecipitation mass spectrometry showed that SMARCC2 and FBXO28 were interrelated (Figure 4A, 4B), and thus, they may interact. The secondary protein profile of FBXO28 is shown in Figure 4C. Co-immunoprecipitation (Co-iP) and immunofluorescence co-localization experiments showed that FBXO28 could bind to SMARCC2 and that FBXO28 and SMARCC2 were mainly localized in the nucleus (Figure 4D, 4E). Moreover, IHC results showed that SMARCC2 was expressed at low levels in PC tissues (Figure 4F, 4G), and FBXO28 was negatively correlated with the expression of SMARCC2 in PC tissues (Figure 4H–4J).

### SMARCC2 upregulation reverses the effect of FBXO28 overexpression

**Figure 5:**
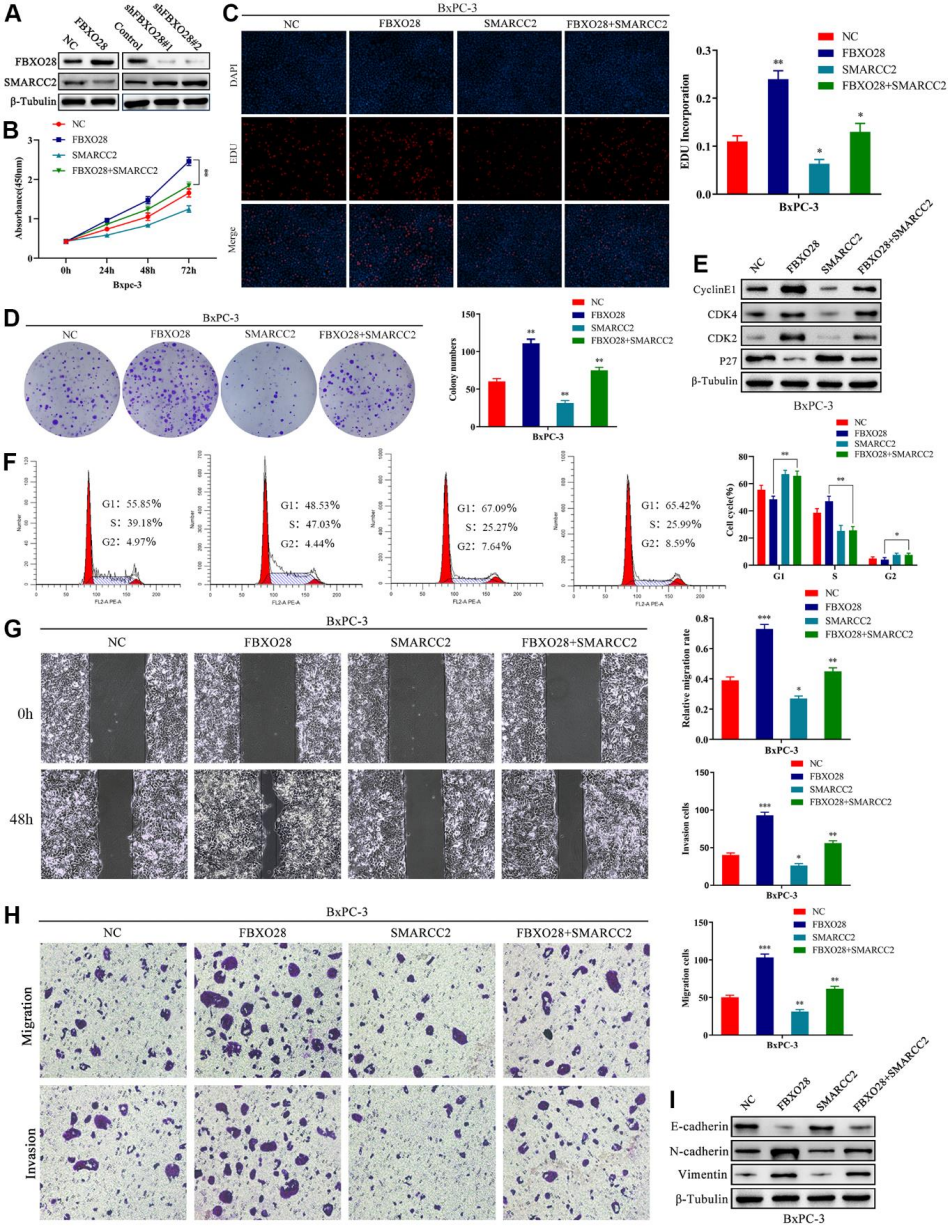
**SMARCC2 upregulation reverses the effect of FBXO28 overexpression.** (**A**) Western blot analysis demonstrating that SMARCC2 expression is lower in cells overexpressing FBXO28 and higher after FBXO28 knockdown. (**B**–**D**) Cell Counting Kit-8 (CCK-8), EdU, and clone plate assays showing that SMARCC2 upregulation inhibits proliferation of FBXO28 overexpression-induced BxPC-3 cells. (**E**, **F**) Western blot and flow cytometry revealing that SMARCC2 upregulation inhibits FBXO28 overexpression-induced BxPC-3 cell cycle. (**G**, **H**) SMARCC2 upregulation inhibited FBXO28 overexpression-induced BxPC-3 cell invasion and migration. (**I**) Western blot to observe the changes in epithelial-mesenchymal transition (EMT)-related proteins in each group following SMARCC2 overexpression. *P < 0.05 **P < 0.01, ***P < 0.001.

According to the results above, we speculated that FBXO28 may have an impact on PC cell proliferation, invasion, and migration by regulating the expression of SMARCC2. The results showed that protein expression of SMARCC2 was decreased or increased after upregulation or downregulation of FBXO28, respectively (Figure 5A), whereas the expression of SMARCC2 mRNA was not affected by FBXO28 (Supplementary Figure 1). To investigate the roles of SMARCC2 and FBXO28, we constructed an upregulated SMARCC2 plasmid and co-transfected it with overexpressed FBXO28. The results of the CCK-8, EdU, clone plate, cell cycle, and western blot tests showed that upregulation of SMARCC2 reduced the proliferative effect of overexpressed FBXO28 to promote BxPC-3 cells (Figure 5B–5F). The wound healing assay, Transwell assay, and western blotting showed that upregulation of SMARCC2 attenuated the effect of overexpressed FBXO28 on promoting the invasion and migration of BxPC-3 cells (Figure 5G–5I). These findings imply that upregulation of SMARCC2 can reverse the effects of overexpressed FBXO28 in promoting the proliferation, invasion, and migration of PC cells.

### FBXO28 regulates SMARCC2 protein expression through ubiquitination

**Figure 6:**
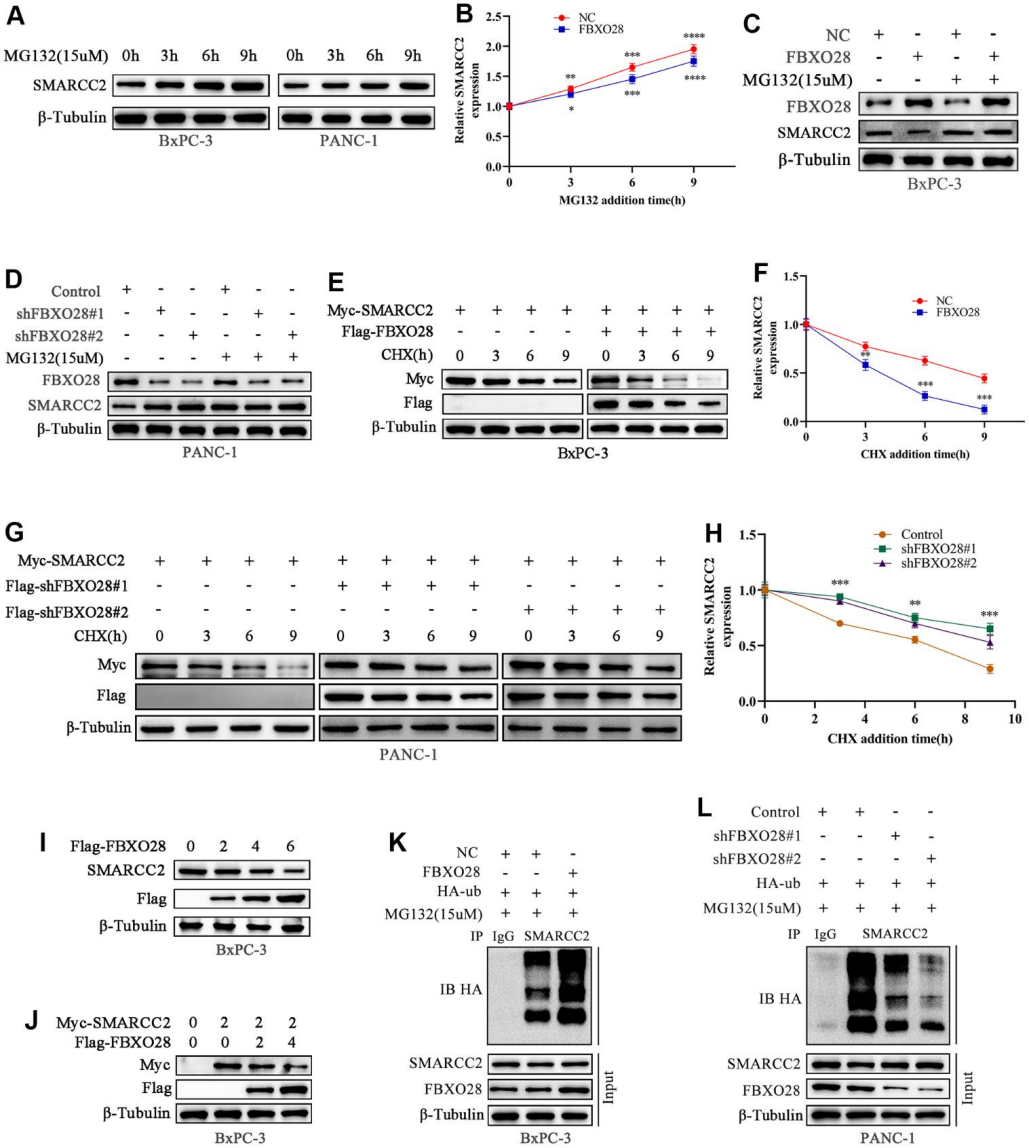
**FBXO28 promotes SMARCC2 ubiquitination.** (**A**, **B**) MG132 (15 μM) was applied to BxPC-3 and PANC-1 cells for the indicated times, and endogenous SMARCC2 levels were detected by western blot. (**C**, **D**) MG132 (15 μM) was added to overexpressing BxPC-3 cells and knockdown PANC-1 cells for the indicated times during the western blot to detect SMARCC2 changes. (**E**–**H**) CHX (20 μM) was applied to BxPC-3 and PANC-1 cells for the indicated times, and western blotting was carried out to detect SMARCC2 degradation. (**I**) BxPC-3 cells were transfected with incremental amounts of Flag-FBXO28 plasmid and detected with anti-SMARCC2 antibody to measure the endogenous SMARCC2 expression level. (**J**) BxPC-3 cells were transfected without treatment or with a single dose of the plasmid encoding Myc-SMARCC2, with or without co-transfection with the increased Flag-FBXO28 plasmid. SMARCC2 expression levels were detected with anti-Myc antibody. (**K**, **L**) Ubiquitin plasmid (HA-ub) plasmids were transfected in overexpressing BxPC-3 cells and knockdown PANC-1 cells, and SMARCC2 protein ubiquitination levels were detected by co-immunoprecipitation. *P < 0.05 **P < 0.01, ***P < 0.001, ****P < 0.0001.

It is unclear whether the ubiquitination ligase FBXO28 regulates SMARCC2 ubiquitination. We treated BxPC-3 and PANC-1 cells with the proteasome inhibitor MG132 which resulted in an incremental increase in endogenous SMARCC2 protein expression, suggesting that the SMARCC2 protein undergoes UPS degradation (Figure 6A, 6B). The addition of MG132 to overexpressed BxPC-3 cells and downregulated PANC-1 cells showed that neither up- nor downregulation of FBXO28 expression significantly affected SMARCC2 protein expression in PC cells (Figure 6C, 6D). Additionally, we added exogenous SMARCC2 plasmids to stably transfected BxPC-3 and PANC-1 cells and treated the cells with CHX at different time points. We found that overexpression of FBXO28 in BxPC-3 cells shortened the half-life of the SMARCC2 protein (Figure 6E, 6F). Conversely, downregulation of FBXO28 in PANC-1 cells prolonged the half-life of the SMARCC2 protein (Figure 6G, 6H). These findings imply that FBXO28 participates in SMARCC2 degradation.

**Figure 7:**
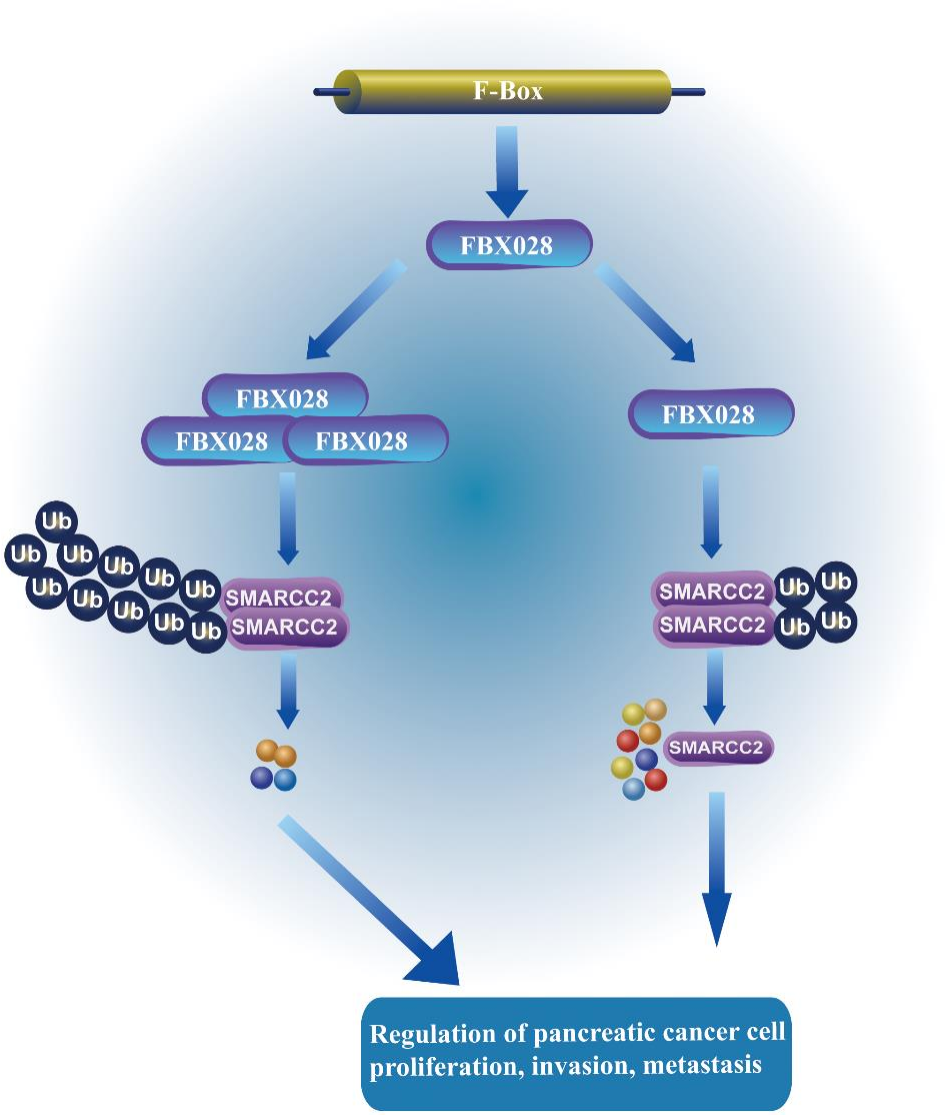
**FBXO28 regulates the mechanism of SMARCC2.** FBXO28 promotes the proliferation, invasion, and metastasis of pancreatic cancer cells by regulating SMARCC2 ubiquitination.

Further evaluation of the role of FBXO28 in SMARCC2 degradation showed that increased levels of exogenous FBXO28 resulted in decreased endogenous SMARCC2 protein expression in BxPC-3 cells (Figure 6I). Using a single dose of Myc-SMARCC2 plasmid and progressively increasing Flag-FBXO28 to co-transfect BxPC-3 cells, we found that exogenous Myc-SMARCC2 decreased with increasing levels of FBXO28 (Figure 6J). These data suggest that FBXO28 promotes SMARCC2 degradation. Finally, we transfected ubiquitin plasmid (HA-ub) in stably expressed BxPC-3 and PANC-1 cells and combined cell lysates with SMARCC2 for immunoprecipitation. We found that overexpression of FBXO28 in BxPC-3 cells increased the ubiquitination level of SMARCC2, whereas downregulation of FBXO28 in PANC-1 cells attenuated the ubiquitination level of SMARCC2 (Figure 6K, 6L). These results suggested that FBXO28 inhibits SMARCC2 expression by ubiquitinating SMARCC2. The study flowchart is shown in Figure 7.

## DISCUSSION

In this study, we identified FBXO28-mediated ubiquitination of SMARCC2 as a key process promoting PC cell growth. We discovered that FBXO28 was highly expressed in PC and verified this by IHC analysis, qRT-PCR, and western blot experiments in PC tissues and cell lines. Tissue microarray analysis of relevant clinical patient pathology data indicated that FBXO28 was correlated with pTNM stage, T stage, and lymphatic metastasis of PC, further verifying that FBXO28 may have an oncogenic function in PC. Functional tests *in vitro* and *in vivo* showed that overexpression of FBXO28 promoted PC cell proliferation, invasion, and metastasis. In contrast, downregulation of FBXO28 inhibited PC cell proliferation, invasion, and metastasis. In addition, we found that SMARCC2 is a potential binding target of FBXO28. We verified that upregulation of SMARCC2 can reverse the effect of FBXO28 on promoting the proliferation, invasion, and metastasis of PC cells through reverse experiments. Finally, using ubiquitination experiments, it was found that FBXO28 promoted the growth of PC by ubiquitinating SMARCC2 and inhibiting its expression. These findings have important implications for novel treatment development, prognostic prediction, and early diagnosis of PC.

Ubiquitination is among the most crucial methods of post-translational modifications; it also regulates different cellular activities by influencing protein stability, translocation, localization, and interactions and participates in almost all biological activities [25, 26]. E3 ligases are the core components of the ubiquitination cascade reaction in which the target specificity of the SCF complex is conferred by F-box proteins, each of which recognizes and binds to a different set of substrates [27]. F-box proteins are generally divided into three subfamilies: FBXW, FBXL, and FBXO [28, 29], with FBXO being the largest subfamily [30]. Increasing evidence shows that FBXO proteins are involved in tumor development as either pro- or anti-oncogenic factors; for example, FBXO4 inhibits breast cancer development by interacting with intercellular adhesion molecule-1 (ICAM-1), promoting its ubiquitination, and decreasing its stability [31]. FBXO22 promotes hepatocellular carcinoma by regulating p21 ubiquitination and degradation [32].

Progression of the cell cycle is regulated by a variety of cyclin-dependent protein kinases, and ubiquitin-mediated cell cycle protein-coupled protein hydrolases and kinase inhibitors play important roles in this process [33, 34]. FBXO28 plays a key role in cell cycle regulation [14, 35]. According to the results of the present investigation, FBXO28 overexpression boosted the expression of Cyclin E1, CDK2, and CDK4 proteins and decreased expression of the P27 protein. In contrast, the opposite results were observed when FBXO28 was knocked down. Mutations and loss of expression of the SWI/SNF complex and its subunits are found in various tumors, such as endometrial cancer [36], esophageal adenocarcinoma [37], lung cancer [38], thyroid cancer [39], and bladder cancer [40]. Tsuda et al. [41] showed that SWI/SNF subunits and their downstream pathways are potential therapeutic targets in PC. SMARCC2, a subunit of the SWI/SNF complex, is known to have a particular function in malignancies. In a previous IHC analysis of 18 surgically resected tumors of pancreatic undifferentiated carcinoma, SMARCC2 expression was not detected in the undifferentiated component in up to 67% of cases [42]. In this study, we demonstrated that FBXO28 interacts with SMARCC2 by Co-iP experiments; the expression of SMARCC2 decreased after overexpression of FBXO28, whereas it increased after knockdown of FBXO28. *In vitro* cell function experiments confirmed that SMARCC2 reversed the effect of FBXO28 overexpression. Collectively, these findings suggest that FBXO28 may promote the proliferation, invasion, and migration of PC cells by regulating the expression of SMARCC2.

Mechanistically, our results confirmed that FBXO28 can participate in the degradation of SMARCC2 via the UPS. The results of ubiquitination experiments showed that the expression of SMARCC2 gradually decreased with increasing exogenous FBXO28. In addition, overexpression of FBXO28 promoted the level of ubiquitination of SMARCC2, whereas knockdown of FBXO28 inhibited SMARCC2 ubiquitination.

## CONCLUSIONS

This study preliminarily found that FBXO28 is highly expressed in PC and is associated with poor patient prognosis. FBXO28 plays a significant role in regulating PC cell proliferation, invasion, and migration. FBXO28 can promote PC cell growth by ubiquitinating SMARCC2 protein expression and, thus, might be a potential treatment target in PC.

## MATERIALS AND METHODS

### Bioinformatics analysis

PC microarray gene expression information was collected from the GEO (https://www.ncbi.nlm.nih.gov/geo/) (GSE15471, GSE62165, and GSE16515) analyzed using R (R Foundation for Statistical Computing, Vienna, Austria). To analyze gene expression in cancer and noncancer samples, Gene Expression Profile Interaction Analysis 2 (GEPIA2, http://gepia2.cancer-pku.cn/) was used, and a boxplot of FBXO28 expression in PC was drawn. RNAseq data (level 3) and accompanying clinical information for PC were collected from The Cancer Genome Atlas (https://portal.gdc.cancer.gov/) database and processed with R. The relationship between the gene and survival prognosis was then analyzed.

### Specimen collection

This study was approved by the Ethics Committee of the Affiliated Hospital of Guizhou Medical University and the Affiliated Cancer Hospital of Guizhou Medical University. Specimens from 26 patients with PC and corresponding paracancerous tissues were obtained from the above units. None of the patients from which tumor specimens were collected had a history of preoperative chemotherapy or radiotherapy. The microarrays of PC tissue were supplied by Shanghai Xinchao Biotechnology Co.

### Cell culture and transfection

The PC cell lines BxPC-3, AsPC-1, MIA PaCa-2, PANC-1, and SW1990 were acquired from the American Type Culture Collection. HPDE, AsPC-1, and BxPC-3 cells were cultivated in RPMI 1640 medium (Gibco, USA); MIA PaCa-2, PANC-1, and SW1990 cells were cultured in Dulbecco’s Modified Eagle Medium (Gibco, USA) supplemented with 10% fetal bovine serum (Gibco, USA) including 1% penicillin and streptomycin. All the cells were cultured at 37° C in a 5% CO_2_ incubator. FBXO28-overexpressing lentiviral vector (FBXO28), FBXO28 shRNA lentiviral vectors (sh-FBXO28#1 and sh-FBXO28#2), negative control lentiviral vectors (control and sh-NC), SMARCC2 plasmid (Myc-SMARCC2), and ubiquitin plasmid (HA-ub) were designed by Shanghai Jikai Biological Co. Lipofectamine 3000 (Invitrogen, USA) was used for transfection, and all steps were carried out in accordance with the instructions.

### RNA extraction and qRT-PCR assay

Total RNA was extracted from PC tissues and cell lines using TRIZOL reagent (Invitrogen, USA). RNA quality and concentration were detected using a NanoDrop spectrophotometer (Thermo Fisher Scientific, USA), and the PrimeScriptTM RT Reagent kit (TaKaRa, Japan) was used for RNA reverse transcription. qRT-PCR analysis was performed using TB Green® Premix Ex TaqTM (Takara, Japan) for FBXO28 (sense 5′-CGAGAACATCCTCAGCTTTATG-3′; antisense 5′-CTCTGGCAGACCAAGTCCAT-3′) and SMARCC2 (sense 5′-AGTGCCAACCCCTTCAC-3′; antisense 5′-GCTCAGGCATCAGGAGAC-3). Glyceraldehyde 3-phosphate dehydrogenase (GAPDH) (sense 5’-CCACAGTCCATGCCATCACTG-3’; antisense 5’-GTCAGGTCCACCACTGACACG-3’) was selected as the endogenous reference.

### Western blot analysis

The total protein of the PC tissues or cell lines was measured using a BCA test (Solarbio, China) after being extracted using RIPA lysis buffer (Merck Millipore, USA). Sodium dodecyl sulfate-polyacrylamide gel electrophoresis (SDS-PAGE) was performed on the samples that were then transferred to polyvinylidene fluoride membranes (0.45 μM, Merck Millipore, USA) and blocked in 5% skim milk for 2 hours. The samples were incubated with the following antibodies: FBXO28 (Sigma-Aldrich, Germany) and SMARCC2, β-tubulin, CDK2, CDK4, P27, E-cadherin, vimentin, N-cadherin, HA, Myc, and Flag (Proteintech, China), overnight at 4° C. After washing three times with Tris-buffered saline with Tween, the corresponding secondary antibody was added, and the sample was incubated for a further 2 hours. Protein expression was detected using electrochemiluminescence imaging (Bio-Rad, USA).

### Cell proliferation assay

The cells were inoculated in 96-well plates at 3×10^3^ cells per well; each group contained three replicate samples. After incubation for 0, 24, 48, and 72 hours, CCK-8 color development solution (GlpBio, USA) was added, and the cells were incubated for 2 hours at 37° C. We used a Quant ELISA Reader (BioTek Instruments, USA) to measure absorption values at 450 nm.

### Colony formation assay

The cells were inoculated at 1×10^3^ per well in a 6-well plate and incubated at 37° C for 2 weeks in a 5% CO_2_ incubator. Thereafter, the cells were fixed with paraformaldehyde, crystal violet staining was performed, and the cells were counted.

### EdU incorporation assay

Following the instructions of the Click-iT EdU-555 kit (Servicebio, China), 20 μM EdU storage solution was added, and the cells were incubated for 2 hours. Subsequently, the cells were fixed in paraformaldehyde and fluorescently stained with iF555 according to the manufacturer’s instructions. Finally, the cells were photographed under a fluorescence microscope (Nikon, Japan), and EdU proliferation-positive cells were expressed as a percentage.

### Flow cytometry assays

The cell cycle staining kit (Servicebio, China) was used according to the manufacturer’s instructions. The cells were extracted and mixed with anhydrous ethanol pre-cooled to -20° C, fixed overnight at 4° C, and washed three times with PBS. Then, 500 μl of the prepared staining solution was added. Thereafter, the cells were incubated at 37° C for 30 minutes in the dark and subjected to flow cytometry (Beckman, USA) followed by cell cycle analysis.

### Wound-healing assays

The cells were seeded in 6-well plates. When the cells reached 100% confluence, they were scratched with a pipette tip to create a wound area at 0, 24, and 48 hours. The wound healing rate was determined using Photoshop (version 2023; Adobe Inc).

### Migration and invasion assays

For the cell transfer experiment in the cell migration and invasion assays, 1×10^4^ cells were seeded into the top chamber of a Transwell plate containing 200 μl of serum-free medium (NEST Biotechnology Co., China), and 800 μl of the medium containing 20% fetal bovine serum was added to the bottom chamber. The untransferred cells at the bottom of the chamber were removed after 24–30 hours of incubation, fixed in 4% paraformaldehyde, stained with crystal violet, and counted under a microscope. A similar method was used for cell invasion examinations, except that Matrigel (R&D Systems, USA) at a concentration of 50 mg/L was added to the upper chamber of the Transwell plate.

### Co-immunoprecipitation and ubiquitination assays

For co-immunoprecipitation, the cells to be tested were extracted, and a cell lysate was prepared. Briefly, 20 μl protein A+G (Beyotime, China) was added to the cell lysate for pre-purification, and the cell lysate was incubated with anti-Flag, anti-SMARCC2, anti-HA, or normal rabbit immunoglobulin G (Proteintech, China) overnight at 4° C. Then, 20 μl protein A+G was added again, and the cells were incubated for 4 hours. The recovered protein-antibody complexes were separated by SDS-PAGE and analyzed by western blot.

For the ubiquitination assay, cells were inoculated in 6-cm dishes and transfected with HA-Ub using Lipofectamine 3000 when the cell density reached 70%–80%. After transfection for 24 hours, 15 μM MG132 (MCE, USA) was added, and the cells were cultured for 9 hours and then extracted for western blot detection of ubiquitination by Co-iP.

### Animal experiments

We separated six-week-old nude mice into five groups of five mice at random and raised them in a particular environment. A subcutaneous injection of 2 × 10^6^ cells was administered in the right axilla of the nude mice from each group. We evaluated the volume of the transplanted tumor every 4 days using vernier calipers and worked out the volume as (length × width^2^)/2. Six weeks after inoculation, the inoculated mice were euthanized; the tumors were removed for weighing, secured in 4% paraformaldehyde, embedded in paraffin, and stained with hematoxylin and eosin. Laboratory practices and animal experiments adhered to the relevant guidelines.

### Statistical analysis

Data are expressed as the mean ± standard deviation. Comparisons between groups were done using the t-test. The Kaplan–Meier method was used for survival analysis. All statistical analyses were performed using GraphPad Prism 8.03. P-values < 0.05 were considered statistically significant.

## Supplementary Material

Supplementary Figure 1
