# Correction for: FBXO28 promotes proliferation, invasion, and metastasis of pancreatic cancer cells through regulation of SMARCC2 ubiquitination

**DOI:** 10.18632/aging.205071

**Published:** 2023-09-14

**Authors:** Songbai Liu, Peng Liu, Changhao Zhu, Rui Yang, Zhiwei He, Yongning Li, Ying Li, Xiaobin Fei, Junyi Hou, Xing Wang, Yaozhen Pan

**Affiliations:** 1 Guizhou Medical University, Guiyan, Guizhou 550000, China; 2 Department of Hepatic-Biliary-Pancreatic Surgery, The Affiliated Cancer Hospital of Guizhou Medical University, Guiyan, Guizhou 550000, China; 3 Department of Hepatic-Biliary-Pancreatic Surgery, The Affiliated Hospital of Guizhou Medical University, Guiyan, Guizhou 550000, China; 4 Department of Hepatobiliary Surgery, Shenzhen Key Laboratory, Shenzhen University General Hospital, Shenzhe, Guangdong 518055, China

**Keywords:** pancreatic cancer, FBXO28, ubiquitination, SMARCC2, proliferation

**This article has been corrected:** The authors found that they incorrectly submitted two images: **Figure 2I** (tumors from the NC and FBXO28 groups), showing upregulation of FBXO28 in BxPC-3 cells in a mouse tumorigenesis experiment, and **Figure 5G** (FBXO28+SMARCC2 48h panel), showing results obtained with transfected BxPC-3 cells in a wound healing experiment. The authors stated that these corrections were prepared using images from the original experiments and do not change the results or conclusions of this paper. The authors apologize for this error.

Corrected **Figure 2** and **5** are presented below.

**Figure 2:**
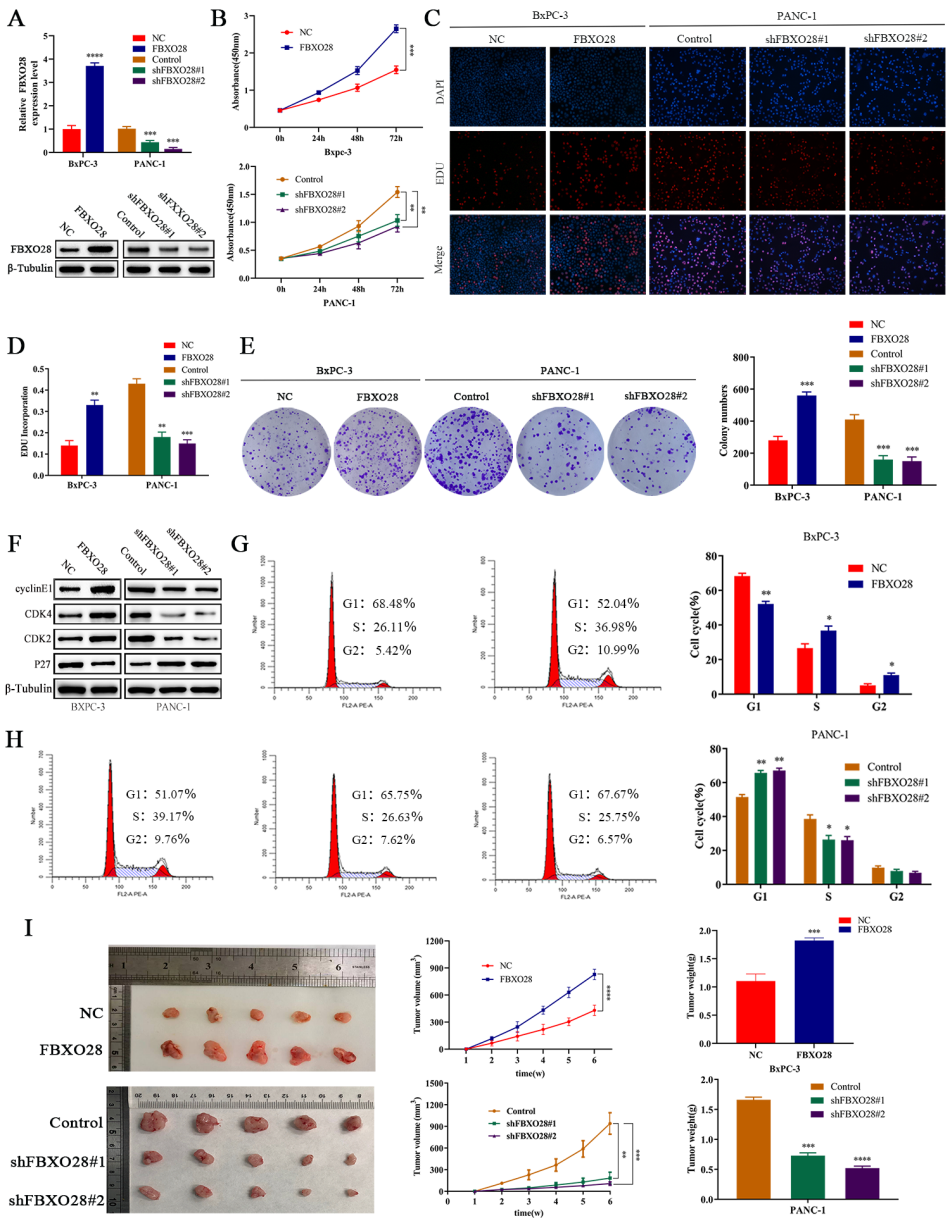
**FBXO28 overexpression increases pancreatic cancer cell proliferation. **(**A**) Lentiviral transfection to form stable cells (negative control [NC], FBXO28, Control, shFBXO28#1, shFBXO28#2) and qRT-PCR and western blot to verify transfection effectiveness.  (**B**–**E**) Cell Counting Kit-8 (CCK-8), EdU, and clone plate experiments were used to identify the capacity of FBXO28 for cell proliferation and formation in pancreatic cancer cells. (**F**–**H**) Western blot and flow cytometry to investigate the effect of FBXO28 on the cell cycle. (**I**) To construct a xenograft model, mice were injected subcutaneously with cells according to grouping, tumor volume was assessed weekly, the mice were euthanized after 6 weeks, and the tumors were resected and weighed. (**J**) Immunohistochemical (IHC) of mouse tumor tissues showing the protein expression of Ki67 and proliferating cell nuclear antigen (PCNA) (magnification: ×200, ×400). **P < 0.01, ***P < 0.001, ****P < 0.0001.

**Figure 5:**
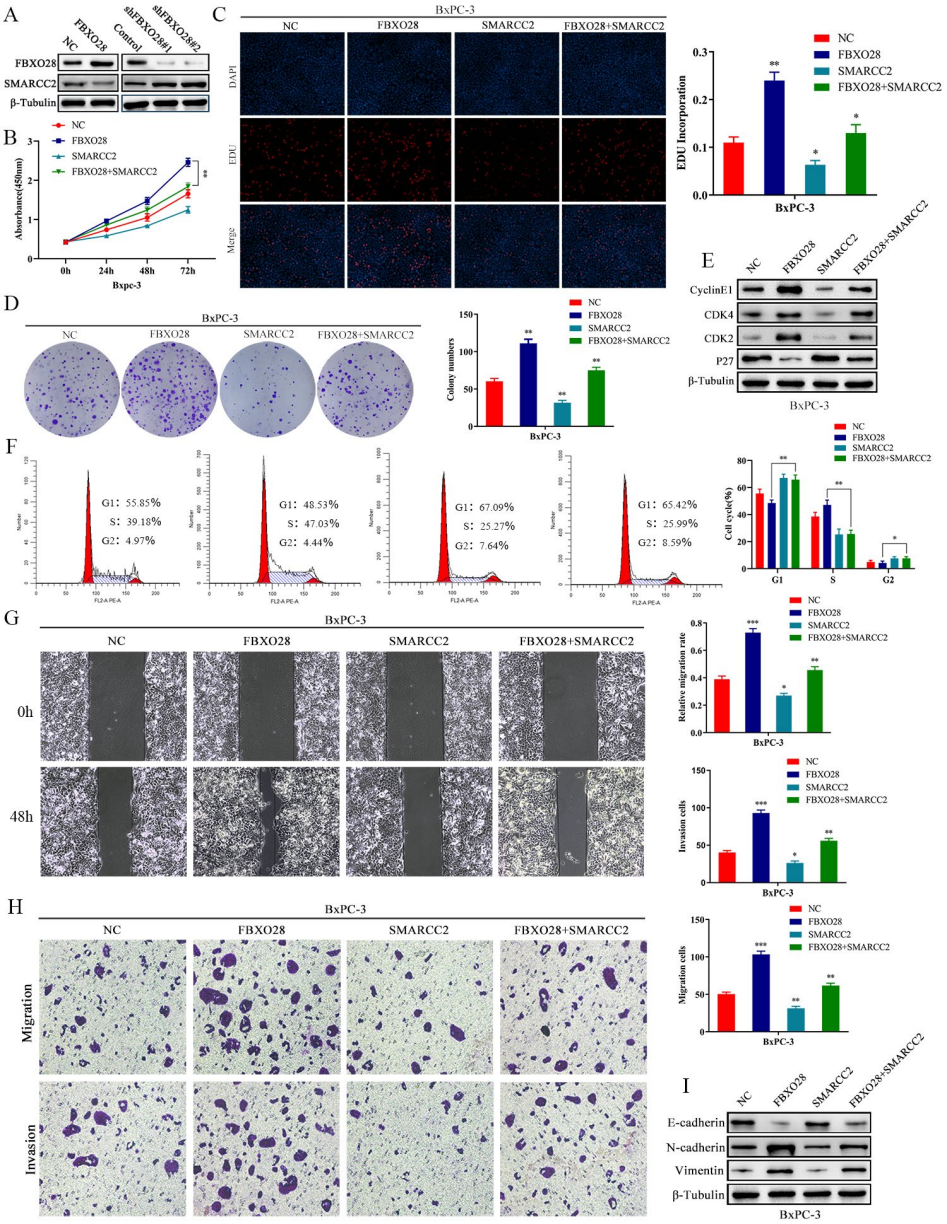
**SMARCC2 upregulation reverses the effect of FBXO28 overexpression. **(**A**) Western blot analysis demonstrating that SMARCC2 expression is lower in cells overexpressing FBXO28 and higher after FBXO28 knockdown. (**B**–**D**) Cell Counting Kit-8 (CCK-8), EdU, and clone plate assays showing that SMARCC2 upregulation inhibits proliferation of FBXO28 overexpression-induced BxPC-3 cells.  (**E, F**) Western blot and flow cytometry revealing that SMARCC2 upregulation inhibits FBXO28 overexpression-induced BxPC-3 cell cycle.  (**G, H**) SMARCC2 upregulation inhibited FBXO28 overexpression-induced BxPC-3 cell invasion and migration. (**I**) Western blot to observe the changes in epithelial-mesenchymal transition (EMT)-related proteins in each group following SMARCC2 overexpression. *P < 0.05 **P < 0.01, ***P < 0.001.

