# Supplementary material for: FBXO28 promotes proliferation, invasion, and metastasis of pancreatic cancer cells through regulation of SMARCC2 ubiquitination
Source: Aging (Albany NY). 2023 Jun 21;15(12):5381–98. doi: 10.18632/aging.204780 (PMC10333084; doi:10.18632/aging.204780)
Supplement: Supplementary Figure 1 [file aging-15-204780-s001.pdf]

SUPPLEMENTARY FIGURE

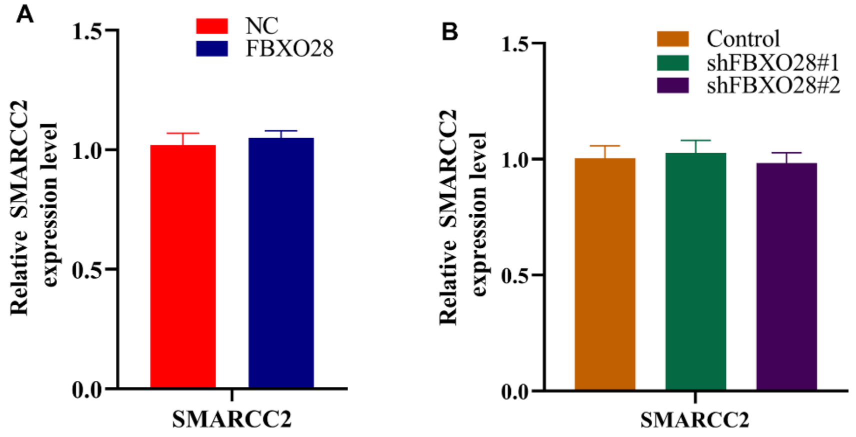

Supplementary Figure 1. Relative SMARCC2 expression level. (**A**, **B**) No significant change in SMARCC2 mRNA expression by overexpression or downregulation of FBXO28.
